# Diffractive Optical Neural Networks: Pathways Toward Intelligent and Scalable Photonic Computing

**DOI:** 10.1002/nap2.70098

**Published:** 2026-04-22

**Authors:** Yufei Wang, Mingheng Zhong, Kun Liao, Quan Sun, Xiaoyong Hu, Qihuang Gong

**Affiliations:** ^1^ State Key Laboratory for Mesoscopic Physics & Department of Physics Collaborative Innovation Center of Quantum Matter & Frontiers Science Center for Nano‐Optoelectronics Peking University Beijing China; ^2^ Key Laboratory for Advanced Optoelectronic Integrated Chips of Jiangsu Province Peking University Yangtze Delta Institute of Optoelectronics Nantong Jiangsu China; ^3^ Collaborative Innovation Center of Extreme Optics Shanxi University Taiyuan Shanxi China; ^4^ Hefei National Laboratory Hefei China

**Keywords:** diffractive optical neural networks, machine learning, multidimensional optical modulation, photonic computing, reconfigurable photonic systems

## Abstract

Optical neural networks have witnessed remarkable progress in recent years, driven by the demand for parallel, high efficiency, and ultrafast information processing. Among various optical neural network architectures, diffractive optical neural networks (DONNs) have emerged as a particularly promising approach due to their scalability in the neuron number, ultrahigh throughput, and passive low‐power operation. Although free‐space implementations laid the foundation for DONNs, on‐chip implementations were later developed to overcome integration challenges and to enhance compatibility with photonic circuits, forming together the two primary platforms of DONNs. Across both free‐space and on‐chip implementations, increasing efforts have been devoted to reconfigurability and multidimensional multiplexing, enabling more flexible and sophisticated optical computing functions. Beyond their intrinsic advantages for imaging and sensing, DONNs have been increasingly adopted for broader neural computing tasks, extending toward large model‐level functionalities. This review provides a comprehensive overview of DONNs, covering their theoretical foundations and structural evolution from free‐space systems to integrated on‐chip and hybrid photonic configurations. It further summarizes recent progress in enhancing computational flexibility through multidimensional modulation and reconfigurable control strategies. Additionally, representative applications and emerging directions are discussed. Finally, the review identifies key challenges and outlines prospective pathways toward intelligent and scalable photonic computing systems.

## Graphical abstract

Diffractive optical neural networks (DONNs) are emerging as a transformative paradigm for photonic computing, offering massive parallelism, ultrafast inference, and passive low‐power operation. This review traces the evolution of DONNs from free‐space to on‐chip implementations, highlights advances in reconfigurability and multidimensional modulation, and outlines pathways toward intelligent, scalable photonic systems capable of large model‐level functionalities.

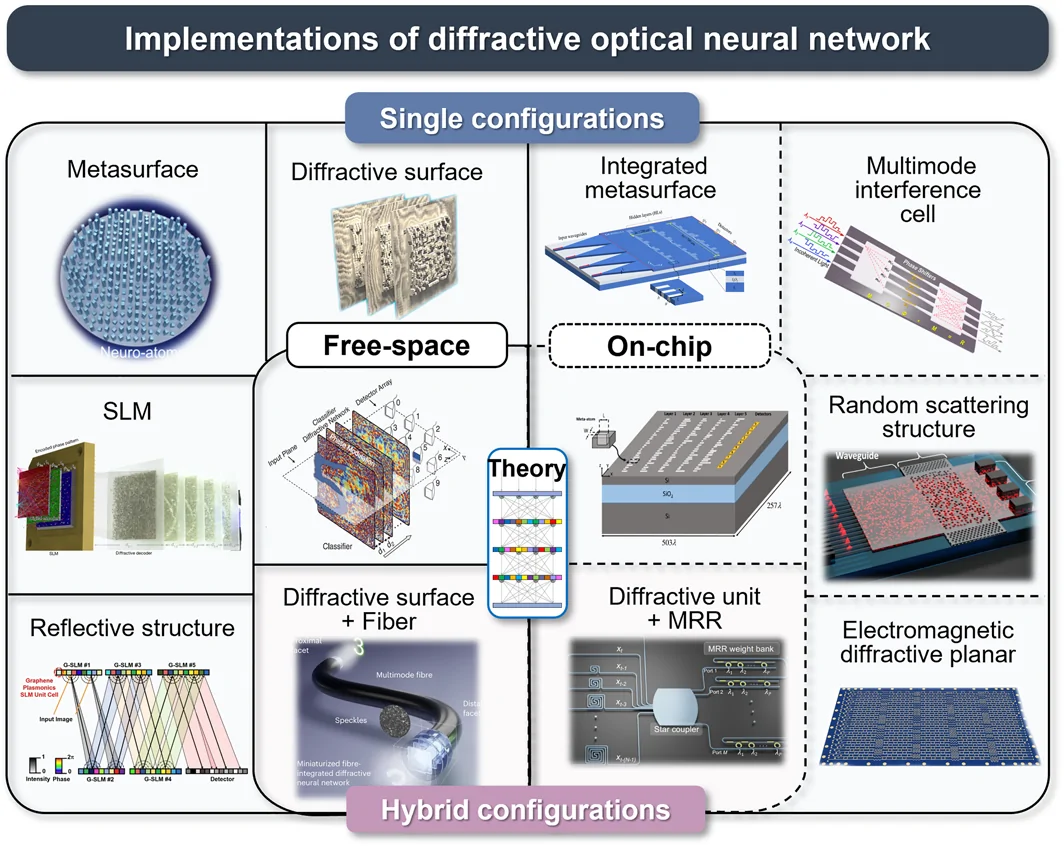

## 1 Introduction

Optical computing has emerged as a powerful paradigm for next‐generation information processing, offering massive parallelism, ultrafast speed, and low power consumption [1, 2]. As the scale and complexity of artificial intelligence models continue to expand, electronic processors are increasingly constrained by bandwidth and energy efficiency [3, 4]. This challenge has revitalized research into optical and photonic computing systems, which can naturally perform high‐throughput operations in a single wave propagation step. In this context, optical neural networks have become an essential bridge between physical optics and artificial intelligence, enabling direct computation in the optical domain [5, 6, 7].

The development of optical neural network architectures has been advancing along three major routes, including Mach–Zehnder interferometer (MZI)–based networks [8, 9, 10, 11, 12], wavelength division multiplexed systems built on microring resonators (MRR) [13, 14, 15, 16, 17], and DONNs [18, 19, 20, 21, 22]. A DONN performs neural computations through diffraction and interference of light across a series of engineered layers, where each layer modulates the amplitude, the phase, and other optical degrees of freedom of the transmitted field, effectively forming a densely connected network processing information in parallel. Compared to MZI‐ and MRR‐based photonic integrated circuits (PICs), DONNs exhibit distinct advantages and limitations. In terms of scalability, owing to their inherently two‐dimensional spatial architecture and compatibility with lithography based or 3D printing fabrication techniques, DONNs offer superior scalability and exceptionally high computational density (A detailed quantitative analysis regarding the physical scalability limits and theoretical upper bounds is provided in the final section.), achieving massive neuron counts that are difficult to attain with cascaded PIC approaches [23]. Regarding reconfigurability, while PICs extensively utilize mature high‐speed electro‐optic modulation [24, 25], DONNs are also evolving beyond static designs. Apart from the widely used slower spatial light modulators (SLMs), various all‐optical and electrically tunable schemes have been developed to enable dynamic adaptability [26, 27, 28, 29], as discussed later in this review. There are differences in computational expressiveness also—PICs are ideal for precise discrete matrix operations, whereas DONNs excel at global feature extraction inherent to wave propagation. Meanwhile, the introduction of nonlinearity is essential for enhanced expressiveness capability. While PICs explore diverse nonlinear schemes, including intrinsic all‐optical material nonlinearities and optoelectronic feedback [30, 31], current DONN architectures primarily realize nonlinear activation through optoelectronic conversion or equivalent nonlinear mappings using linear materials [32, 33, 34]. Furthermore, regarding integration, unlike PICs that benefit from mature planar packaging, free‐space implementations of DONNs often face challenges in alignment and miniaturization, and on‐chip implementations have achieved compact footprints and demonstrated excellent compatibility with established photonic integration platforms. These characteristics collectively make DONNs a promising platform for optical computing [35].

As illustrated in Figure 1, the development of DONN architectures can be broadly categorized into two main classes: free‐space and on‐chip implementations. The concept of the DONN was first realized in free‐space systems [18], where diffractive layers were physically constructed using 3D‐printed diffractive surfaces. Free‐space implementations excel at large‐scale optical processing and computation, providing direct access to the encoded optical information in 3D [40]. However, their large physical footprint and sensitivity to optical alignment impose practical limits on integration. To address these challenges, on‐chip implementations have been developed as a complementary branch [37, 41, 42], offering higher integration density and improved compatibility with existing photonic and electronic platforms. Together, free‐space and on‐chip implementations now form the two primary platforms of DONNs, each offering distinct advantages in scalability, compactness, and functionality.

**FIGURE 1:**
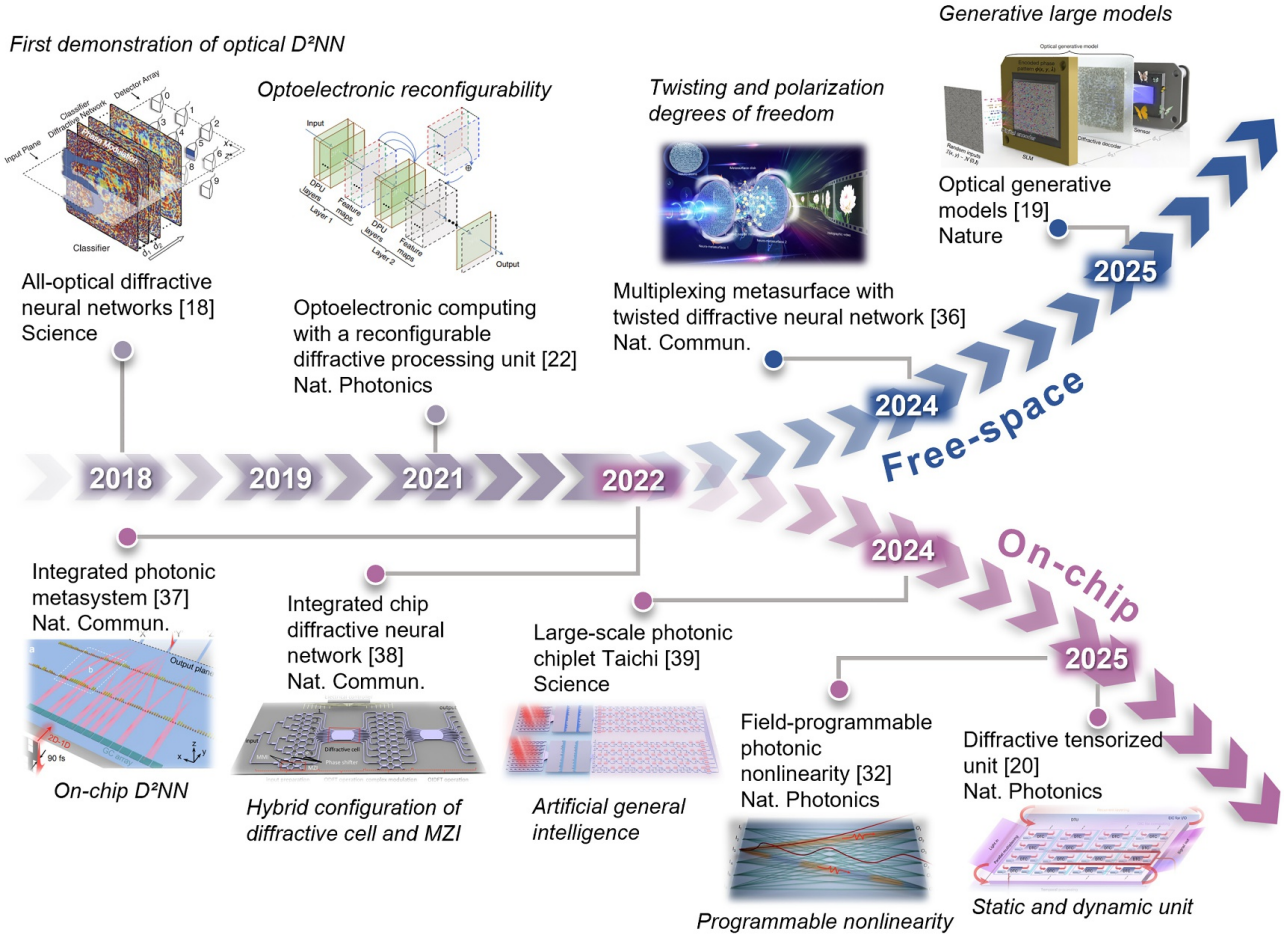
Evolution of DONNs from the initial concept to the establishment of free‐space and on‐chip branches. All‐optical diffractive neural networks. Reprinted with permission from Ref. [18]. Copyright 2018, The American Association for the Advancement of Science. Optoelectronic computing with a reconfigurable diffractive processing unit. Reprinted with permission from Ref. [22]. Copyright 2021, Springer Nature. Multiplexing metasurface with a twisted diffractive neural network. Reprinted with permission from Ref. [36]. Copyright 2024, Springer Nature. Optical generative models. Reprinted with permission from Ref. [19]. Copyright 2025, Springer Nature. Integrated photonic metasystem. Reprinted with permission from Ref. [37]. Copyright 2020, Springer Nature. Integrated chip diffractive neural network. Reprinted with permission from Ref. [38]. Copyright 2022, Springer Nature. Large‐scale photonic chiplet Taichi. Reprinted with permission from Ref. [39]. Copyright 2024, The American Association for the Advancement of Science. Field‐programmable photonic nonlinearity. Reprinted with permission from Ref. [32]. Copyright 2025, Springer Nature. Diffractive tensorized unit. Reprinted with permission from Ref. [20]. Copyright 2025, Springer Nature.

Beyond structural innovation, enhancing the computational capabilities of DONNs has become a central direction in their continued development [43]. These capabilities ultimately determine the range and complexity of tasks that a network can accomplish, and they are largely shaped by two key factors: reconfigurability and multidimensional modulation. As shown in Figure 1, both free‐space and on‐chip implementations have advanced along these key factors. Static optical layers, while efficient, lack the tunability required for dynamic or multitask operations. To overcome this limitation, reconfigurable mechanisms have been introduced, including thermo‐optic, electro‐optic [20, 29, 44], and all optical modulation in on‐chip implementations [26, 32], as well as SLMs or liquid crystals (LCs) based programmable surface and other electrically controlled meta‐atoms in free‐space implementations [19, 21, 22, 27, 28, 33, 34, 45, 46, 47, 48, 49]. In parallel, multidimensional modulation strategies leveraging multiple optical degrees of freedom [27, 28, 33, 34, 36, 37, 45, 48, 49, 50, 51, 52, 53, 54, 55, 56, 57, 58, 59, 60, 61, 62, 63, 64, 65], such as polarization, wavelength, amplitude, phase, orbital angular momentum (OAM), and spatiotemporal multiplexing, further enrich the versatility of DONNs. Collectively, these advances are charting the pathways toward intelligent and scalable photonic computing.

In terms of functionality, DONNs were first demonstrated for classical imaging and classification tasks [18, 66, 67, 68], where they provided efficient, low‐latency solutions for optical inference. With the continuing evolution of optical design and computational algorithms, their applications have expanded to include beam shaping [69], encryption [50, 52, 55], communication [27, 28, 67], mathematical computation, and even generative large models’ processing [20, 39]. The growing diversity of DONN applications highlights their potential to bridge physical optics and intelligent computation.

Despite these advances, several key challenges remain. The integration of nonlinear operations, in situ training, stability under fabrication variations, and scalability of complex architectures continue to limit the full potential of DONNs. This review provides a systematic and forward‐looking overview of DONNs. We first discuss the theoretical principles and physical foundations of diffractive computing, followed by the practical implementations of DONNs, including free‐space, on‐chip, and hybrid configurations. We then summarize recent progress in reconfigurable control and multidimensional modulation, highlighting key mechanisms that enhance computational capabilities. Finally, we review representative applications and outline the current challenges and future prospects for realizing intelligent and scalable photonic computing systems.

## 2 Theoretical Framework and Practical Implementations of DONNs

A proliferation of proposals for various DONN configurations has been witnessed in recent years. The broad spectrum of these configurations can be divided into free‐space and on‐chip implementations, as presented in the left and right sections of Figure 2, respectively. The free‐space implementation of DONNs primarily encompasses transmissive configurations such as diffractive surfaces [27], metasurfaces [36], and SLMs [19], alongside reflective architectures [70]. In contrast, on‐chip implementations typically involve integrated metasurfaces [42], multimode interference (MMI) cells [72], random scattering structures [73], and electromagnetic diffractive planars [74]. In addition to single configurations, DONNs can also be integrated with other architectures such as fibers [65] and microring resonators [38] to form multifunctional hybrid configurations. Representative examples of each category, along with their schematic illustrations, are also provided in Figure 2.

**FIGURE 2:**
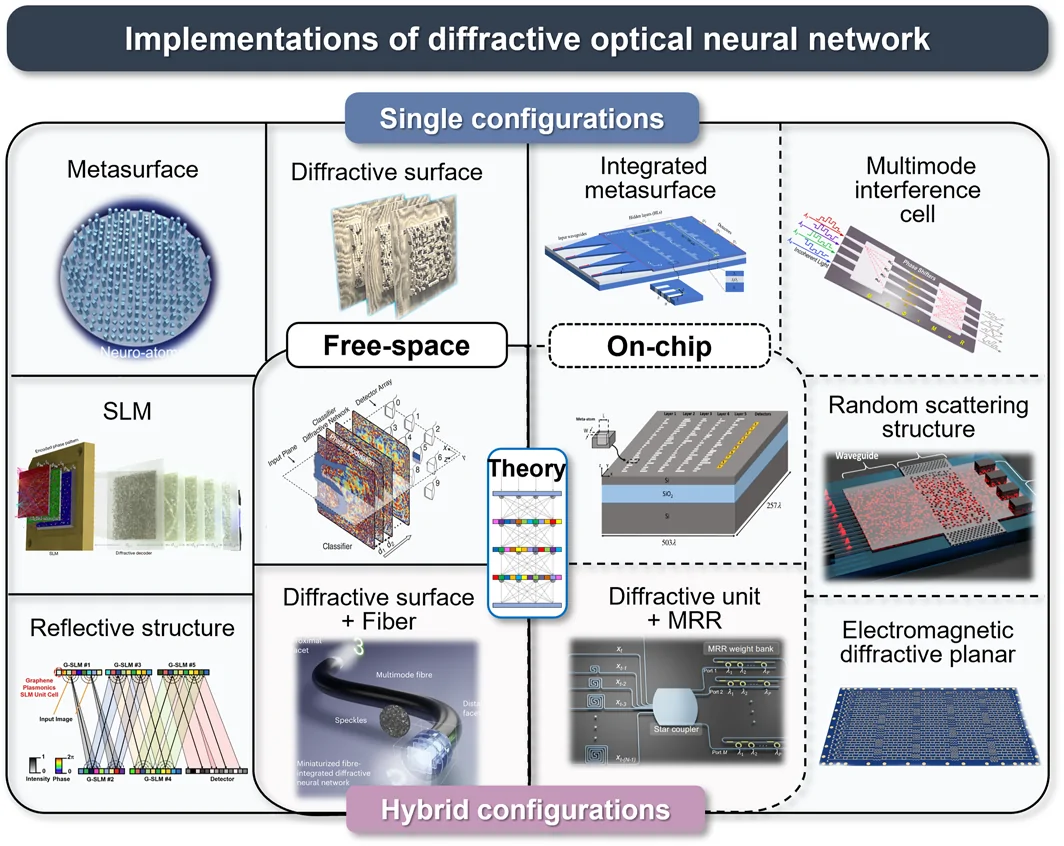
Overview of DONN implementations comprising single configurations (free‐space and on‐chip) and hybrid configurations. The configuration of free‐space implementations is represented. Reprinted with permission from Ref. [18]. Copyright 2018, The American Association for the Advancement of Science. Metasurface. Reprinted with permission from Ref. [36]. Copyright 2024, Springer Nature. Diffractive surface. Reprinted with permission from Ref. [27]. Copyright 2024, Springer Nature. SLM. Reprinted with permission from Ref. [19]. Copyright 2025, Springer Nature. Reflective structure. Reprinted with permission from Ref. [70]. Under License CC BY 4.0. The configuration of on‐chip implementations is represented. Reprinted with permission from Ref. [71]. Copyright 2025, Springer Nature. Integrated metasurface. Reprinted with permission from Ref. [42]. Copyright 2023, Springer Nature. Multimode interference cell. Reprinted with permission from Ref. [72]. Copyright 2023, Springer Nature. Random scattering structure. Reprinted with permission from Ref. [73]. Copyright 2024, Springer Nature. Electromagnetic diffractive planar. Reprinted with permission from Ref. [74]. Copyright 2024, The American Association for the Advancement of Science. Diffractive surface + fiber. Reprinted with permission from Ref. [65]. Copyright 2025, Springer Nature. Diffractive unit + MRR. Reprinted with permission from Ref. [75]. Copyright 2024, Springer Nature.

Despite their apparent diversity, these configurations universally adhere to the fundamental structure of a neural network and can be described by a unified theoretical framework for physical computation. Prior to a detailed discussion of these configurations, it is essential to introduce their theoretical principles.

### 2.1 Theoretical Principles of DONNs

Based on the principle of diffraction, wave fronts originating from a light source propagate and spatially expand. This physical mechanism enables the establishment of interconnections among neurons: Light arriving at any point is the result of waves originating from multiple locations, and light emitted from a single point can illuminate an extended area. Similar to an artificial neural network, a DONN also comprises an input layer, hidden layers, and an output layer. Light emitted from the input layer carries the initial information. By modulating the amplitude and/or phase of the light field as it propagates through these diffractive layers, neuron‐like functionality is achieved. The processed information is then read out at the output layer, thereby completing the computational process. The following section outlines the fundamental methodology for such modeling.

Consider a DONN architecture comprising m hidden layers, all oriented perpendicular to the optical axis (*z*‐axis). Under coherent illumination, an input wave front is normally incident on the input plane and propagates sequentially through the hidden layers along the positive *z*‐direction before reaching the output plane. The optical field at the input layer can be mathematically represented by a complex amplitude distribution denoted as

(1) $$\begin{array}{c} u_{0}^{\text{out}}\left(r_{0}\right)=I\left(x_{0},y_{0}\right) \end{array},$$

where $r_{0}=\left(x_{0},y_{0},z_{0}\right)$ denotes the coordinates on the input plane. For the ith hidden layer, the relationship between its input and output optical fields is governed by

(2) $$\begin{array}{c} u_{i}^{\text{out}}\left(r_{i}\right)=f_{i}\left(u_{i}^{\text{in}};r_{i}\right) \end{array},$$

In this expression, $f_{i}$ denotes the modulation applied at position $r_{i}$ on the ith hidden layer. Typically, this modulation is linear and can be expressed in terms of a complex transmittance:

(3) $$\begin{array}{c} u_{i}^{\text{out}}\left(r_{i}\right)=t_{i}\left(r_{i}\right)\cdot u_{i}^{\text{in}}\left(r_{i}\right) \end{array},$$

in which the transmittance is a complex function including changes in amplitude $a_{i}\left(r_{i}\right)$ and phase $\phi_{i}\left(r_{i}\right)$:

(4) $$\begin{array}{c} t_{i}\left(r_{i}\right)=a_{i}\left(r_{i}\right)e^{j\phi_{i}\left(r_{i}\right)} \end{array},$$

with j being the imaginary unit. The propagation of light between two layers can be described by the Rayleigh–Sommerfeld diffraction integral as follows:

(5) $$\begin{array}{c} u_{i+1}^{\text{in}}\left(r_{i+1}\right)=\iint u_{i}^{\text{out}}\left(r_{i}\right)\cdot h\left(r_{i};r_{i+1}\right)dx_{i}dy_{i} \end{array}.$$

The integral is performed on the ith layer. In this formula, $h\left(r_{i};r_{i+1}\right)$ is the diffraction response function between the ith and (i+1)th layers, having the following expression:

(6) $$\begin{array}{c} h\left(r_{i};r_{i+1}\right)=\frac{z_{i+1}-z_{i}}{2\pi R^{2}}\cdot \left(\frac{1}{2\pi R}+\frac{1}{j\lambda }\right)\cdot e^{j\frac{2\pi R}{\lambda }} \end{array},$$

where $R=|r_{i+1}-r_{i}|=\sqrt{{\left(x_{i+1}-x_{i}\right)}^{2}+{\left(y_{i+1}-y_{i}\right)}^{2}+{\left(y_{i+1}-z_{i}\right)}^{2}}$, and λ represents the wavelength of light. The optical plane is typically discretized into a large number of small, distinct regions, each referred to as a neuron. When the size of these neurons is sufficiently small relative to the propagation distance R, the Rayleigh–Sommerfeld integral can be approximated by a discrete summation as follows:

(7) $$\begin{array}{c} u_{i+1}^{\text{in}}\left(r_{i+1}\right)=\sum_{k=1}^{N}u_{i}^{\text{out}}\left(r_{i,k}\right)\cdot h\left(r_{i,k};r_{i+1}\right)\Delta x_{i,k}\Delta y_{i,k} \end{array},$$

in which N is the number of neurons in the layer. Substituting the previous linear approximation, we get the transformation relationship for forward propagation through one layer as follows:

(8) $$\begin{array}{c} u_{i+1}^{\text{in}}\left(r_{i+1}\right)=\sum_{k}\left[t_{i}\left(r_{i}\right)\cdot h\left(r_{i};r_{i+1}\right)\Delta x_{i}\Delta y_{i}\right]\cdot u_{i}^{\text{in}}\left(r_{i}\right) \end{array}.$$

The light field is converted into a measurable signal at the output layer. When a light intensity detector is employed, the output is obtained as the measured optical intensity as follows:

(9) $$\begin{array}{c} s\left(r_{m+1}\right)={\left|u_{m+1}^{\text{in}}\left(r_{m+1}\right)\right|}^{2} \end{array}$$

Based on the established theoretical and modeling principles, the design space of DONNs can be systematically explored. Building upon this foundation, we now turn to a detailed discussion of the primary DONN configurations categorized into free‐space, on‐chip, and hybrid configurations, each offering distinct advantages and implementation considerations that will be examined in the following sections.

### 2.2 Free‐Space Implementations

The initial implementation of a DONN was pioneered by Lin et al. in a free‐space implementation [18]. By directly controlling the optical wave front, free‐space DONNs facilitate the processing of 2D or even 3D information while utilizing more degrees of freedom of light. They also exhibit high reconfigurability, permitting their realization through diverse structural forms. Figure 3 depicts several representative free‐space implementations that have emerged.

**FIGURE 3:**
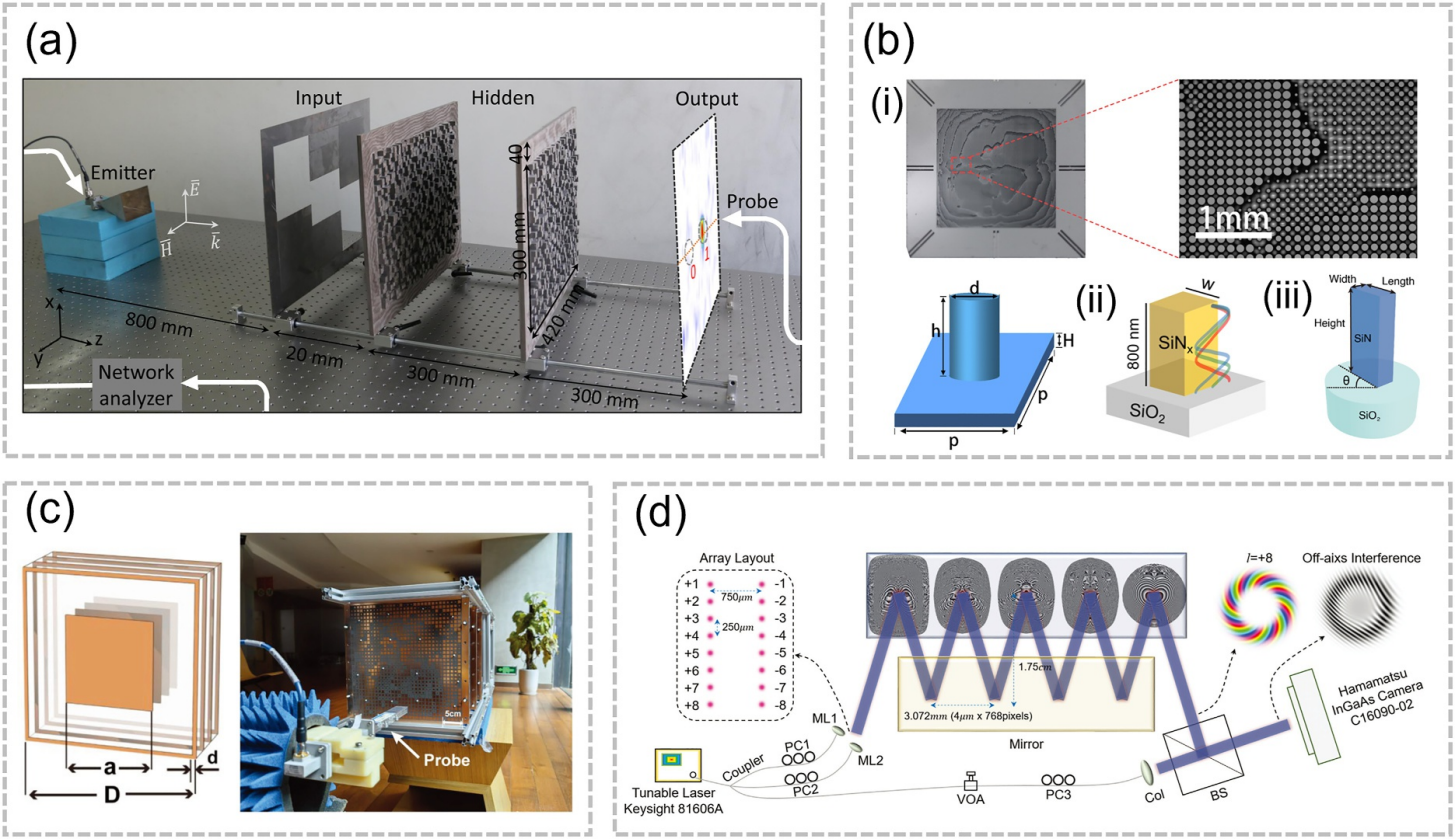
Free‐space implementations of DONN. (a) A free‐space DONN realized with cascaded diffractive surfaces, where phase modulation is introduced via spatially varying thickness profiles to implement trainable wave front transformation through free‐space diffraction, enabling optical logic and decision‐making functionalities. Reprinted with permission from Ref. [76]. Copyright 2020, Springer Nature. (b) Transmissive metasurface‐based DONN architectures employing different meta‐atom geometries for phase control, including (i) cylindrical nanopillars with radius‐dependent phase response. Reprinted with permission from Ref. [77]. Under License CC BY 3.0. (ii) Rectangular nanopillars with width‐tuned phase modulation. Reprinted with permission from Ref. [78]. Copyright 2025, Springer Nature. (iii) Meta‐atoms with rotation‐controlled phase. Reprinted with permission from Ref. [50]. Copyright 2023, Springer Nature. (c) A microwave DONN implemented using metallic meta‐units, where phase shifts are achieved by tuning patch geometries. The left panel shows the schematic of the meta‐unit structure, whereas the right panel presents the experimental configuration of multilayer diffractive networks, demonstrating free‐space inference in the microwave regime. Reprinted with permission from Ref. [79]. Copyright 2024, John Wiley. (d) A reflective free‐space DONN architecture based on cascaded phase plates serves as the central computational module, embedded within an experimental setup for OAM multiplexing and complex field measurement using off‐axis interference. Reprinted with permission from Ref. [80]. Copyright 2023, WILEY ‐ VCH.

As illustrated in Figure 3a [76], the diffractive surface represents the most fundamental configuration. Each layer contains a large number of individual units serving as neurons, whose feature size is generally not smaller than the order of the wavelength. Depending on the unit's thickness $h_{i}$ and the refractive index of the material and the surrounding medium, $n_{i}$ and $n_{0}$ respectively, the phase shift imparted to the transmitted light can be expressed as follows:

(10) $$\begin{array}{c} \phi_{i}=\frac{2\pi }{\lambda }\left(n_{i}-n_{0}\right)h_{i} \end{array}.$$

Materials with high transmittance are typically preferred, and by modulating either the thickness or the refractive index of these units, the functionality of a hidden layer can be effectively realized. Gao et al. employed a material with high transmittance and a stable refractive index [27], and the diffractive plane was fabricated by spatially varying its thickness. As demonstrated by Ocier et al.'s attempt [81], varying the refractive index offers an alternative approach for implementing the diffractive surfaces.

However, a fundamental constraint in this configuration is that the neuron size is typically comparable to the operating wavelength. To reduce spatial footprint and achieve enhanced light manipulation, metasurfaces composed of subwavelength dielectric pillars have been widely adopted. These meta‐atoms, with dimensions substantially smaller than the wavelength, enable precise phase modulation through variations in their geometric parameters or orientations. As illustrated in Figure 3bi, Liu et al. utilized cylindrical nanopillars with gradually altered cross‐sectional radii to impose phase shifts [77]. Similarly, Figure 3bii and iii depict square nanopillars that modulate the phases by adjusting the side width [78] and the in‐plane rotation angle [50], respectively. In addition to phase modulation, such metasurface architectures can also manipulate other degrees of freedom of light, such as OAM [45], polarization [36], and direction [55], along with additional optical dimensions that will be discussed in detail in the following sections.

Metals also serve as effective components for modulation, owing to their strong electromagnetic response in the microwave regime. As shown in Figure 3c, the phase shift of a metallic meta‐atom can be tailored via width a [79]. Furthermore, metallic Huygens' metasurfaces have been employed to construct DONNs [82]. Leveraging the high conductivity of metals, reconfigurable intelligent surfaces (RIS) integrated with field‐programmable gate arrays (FPGAs) further enable programmable DONN systems [28], which allows independent control over the transmittance of each neuron.

The aforementioned configurations primarily employ forward‐propagating transmission schemes. An alternative approach utilizes reflective architectures to implement DONNs. In such designs, light interacts with the diffractive layers at an oblique angle, with neural functionality being encoded through phase or amplitude modifications upon reflection at each interface. As illustrated in Figure 3d, a reflective DONN may be constructed using mirrors to optically link successive layers [80]. Furthermore, graphene‐plasmonic reflective spatial light modulators have been proposed as a platform for reconfigurable DONN implementations [70]. Beyond leveraging unique wave–matter interactions in reflective geometries, this architecture offers a significantly reduced space occupation. A notable example includes an on‐chip integration of reflective hidden layers using compact planar waveguide circuits [83]. The rich diversity of free‐space implementations opens up possibilities for exploring a wider range of functionalities and applications.

### 2.3 On‐Chip Implementations

In addition to free‐space implementations, significant research efforts have been increasingly directed toward on‐chip implementations, with remarkable progress achieved in recent years [20, 37, 73, 84]. On‐chip DONNs offer compact form factors, compatibility with modern electronic chip ecosystems, and scalability for executing large‐scale computational tasks. Several representative on‐chip architectures are illustrated in Figure 4.

**FIGURE 4:**
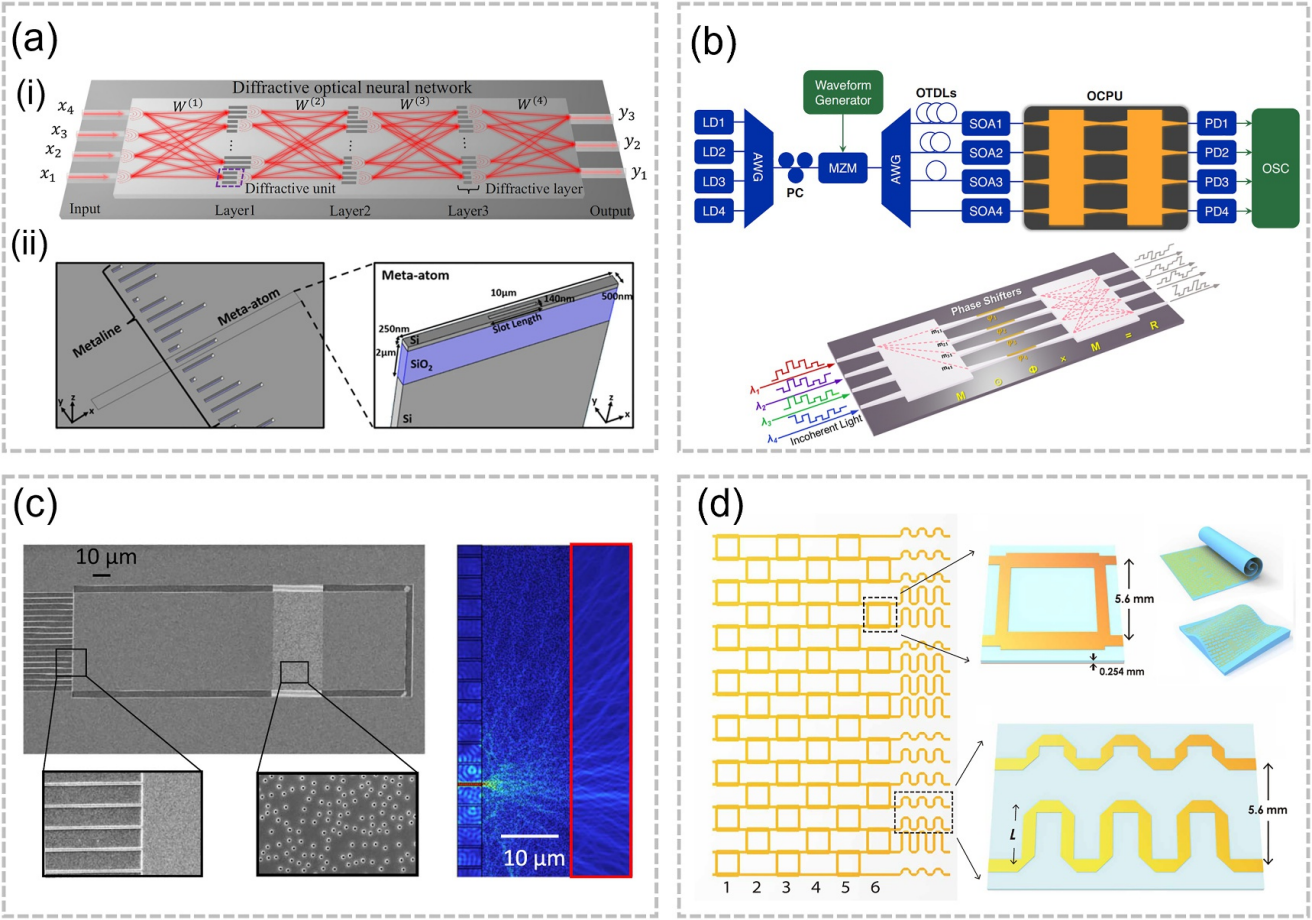
On‐chip implementations of DONNs. (a) Integrated metasurface composed of meta‐atom–based metalines, implemented as etched slots on a silicon substrate. Each diffractive unit on a given layer acts as a secondary wave source, the amplitude and phase of which are determined by the product of the input wave and the complex‐valued transmission at that unit. (i) Neuron interconnection architecture. Reprinted with permission from Ref. [42]. Copyright 2023, Springer Nature. (ii) Meta‐atom array configuration. Reprinted with permission from Ref. [85]. Copyright 2022, Springer Nature. (b) The upper panel illustrates the experimental setup for convolution computing based on an optical convolution processing unit (OCPU), whereas the lower panel depicts the OCPU structure, comprising two 4 × 4 multimode interference cells and four phase shifters. Reprinted with permission from Ref. [72]. Copyright 2023, Springer Nature. (c) The left panel illustrates an SEM image of the fabricated silicon photonic encoder, illustrating the integrated waveguide architecture and the compact scattering region that enables random optical encoding on chip. The right panel shows full‐wave numerical simulations of the optical encoder. Reprinted with permission from Ref. [73]. Copyright 2024, Springer Nature. (d) Electromagnetic diffractive planar structure for guided wave manipulation, including the printed circuit layout of the propagation and phase modulation layers, which are shown in enlarged views on the right side of the figure. Reprinted with permission from Ref. [74]. Copyright 2024, The American Association for the Advancement of Science.

The integrated metasurface represents one of the earliest adopted on‐chip implementations for DONNs. Figure 4ai depicts the interconnecting architecture between neural layers [42]. As shown in Figure 4aii, each hidden layer is implemented as a metaline consisting of meta‐atoms [85]. Each meta‐atom takes the form of an etched slot on a silicon substrate, functioning as an individual neuron. The transmitted amplitude and phase delay of these slots are fully controllable through precise adjustments to their geometric parameters, including width, length, and etching depth. This configuration has been widely employed across numerous studies and has demonstrated versatility in accomplishing various computational tasks [54, 56, 71, 86].

Hidden layers can also be realized using MMI cells, known for their flexible tunability and inherent support for multimode operation. Figure 4b illustrates the working principle of an MMI‐based design as demonstrated in Reference [72]. By integrating TiN heaters for localized thermal tuning of individual neurons, Cheng et al. further introduced a configuration capable of in situ training [29]. Additionally, this architecture can be augmented with nonlinear layers fabricated from germanium, as shown in Reference [44], thereby significantly enhancing its computational capability. Tunable MMI devices hold significant potential for the development of reconfigurable on‐chip DONNs.

All the aforementioned architectures share a common characteristic: the absence of structural confinement between successive layers, permitting light to undergo free diffraction. As illustrated in Figure 4c, a random encoder design was introduced in Reference [73] for optical image compression, where the scattering structure inherently assists in performing optical convolution. Meanwhile, Figure 4d depicts an electromagnetic diffractive planar structure that guides microwaves along a confined path, enabling robust computation without misalignment [74]. If printed on the flexible substrate, it could be rolled up in the spiral structure. Building upon these on‐chip implementations, novel architectures and application scenarios continue to emerge.

### 2.4 Hybrid and Compound Configurations

To address diverse objectives, DONNs have been successfully implemented in hybrid and compound configurations, where they are integrated with complementary electronic or optical systems to achieve advanced functionalities.

Optical fibers play a pivotal role in modern communications. Integrating a DONN at the distal facet of a multimode fiber enables direct all‐optical image transmission, providing unprecedented functionalities [65]. Fiber‐based DONNs have also demonstrated high performances in various machine learning tasks [87]. These advances highlight the potential of fiber‐integrated diffractive systems to serve as compact and efficient platforms for communications and artificial intelligence.

Diffractive units have been widely integrated into photonic chips alongside conventional components such as MZIs and microring resonators. For instance, by employing compact diffractive cells, the number of MZIs required to process N inputs can be reduced from $N^{2}$ to N [38]. In a notable hybrid design combining diffractive and interference‐based processing within a general distributed computing architecture, a photonic chiplet was demonstrated with millions of neurons’ capacity and an energy efficiency of 160 tera‐operations per second per watt [39]. Diffractive structures have also facilitated signal mixing in ultrafast reservoir computing systems [75]. More recently, a fully reconfigurable photonic processor based on diffractive units has been proposed [20]. These developments collectively affirm the efficacy of diffractive elements is due to their efficient interconnections and processing units in hybrid optical neural networks.

To provide a clearer comparison between different DONN implementation platforms, Table 1 summarizes the key performance metrics of representative free‐space and on‐chip DONN systems reported in recent years.

**TABLE 1:** Quantitative comparison of representative free‐space and on‐chip DONN implementations.

Platform	Ref.	Architecture	Layers	Neuron count^a^	Throughput (input channel × output channel)	Footprint^b^ (each layer)	Power
Free‐space	Lin et al. Science, 2018 [18]	3D‐printed diffractive surfaces	5	∼0.2 M	200 × 200	8 × 8 cm^2^	Passive for the diffractive module
Free‐space	Zhou et al. Nat. Photonics, 2021 [22]	DMD‐based (D‐RNN)	3	∼1.47 M	700 × 700	6.44 × 6.44 mm^2^	71.4 W (CMOS + DMD + SLM + source)
Free‐space	Liu et al. Nat. Electron., 2022 [28]	Digital coding metasurface	5	320	8 × 8	560 × 560 mm^2^	∼100 W (FPGA + metasurface)
Free‐space	Zheng et al. Nat. Mach. Intell., 2023 [21]	SLM‐based dual adaptive training (DPNN‐C)	3	∼0.12 M	200 × 200	17 × 17 μm^2^	163.7 mW (source)
Free‐space	Yildirim et al. Nat. Photonics, 2024 [34]	SLM + mirror	4	∼0.36 M	300 × 300	2.4 × 2.4 mm^2^	/
Free‐space	Chen et al. Science, 2025 [19]	Metasurfaces + single‐mode fiber array	6	∼2 M	512 × 512	1.8 × 1.8 mm^2^	Passive for the diffractive module 53.85 W (SLM + source + camera)
On‐chip	Zhu et al. Nat. Commun., 2022 [38]	Integrated diffractive cell	2	200^b^	10 × 10	0.04 mm^2^	Passive for the diffractive module
On‐chip	Fu et al. Nat. Commun., 2023 [42]	Si metaline integrated DONNs	3	558 (iris task)	4 × 3	280 × 250 μm^2^	Passive for the diffractive module
On‐chip	Wu et al. Nat. Photonics, 2023 [26]	Lithography‐free InGaAsP slab	1	32^b^	8 × 4	500 × 324 μm^2^	∼1.5 mW (optical pumping)
On‐chip	Meng et al. Nat. Commun., 2023 [72]	MMI‐based convolution unit	2	32^b^	4 × 4	275 × 15 μm^2^	∼100 mW (thermo‐optic phase shifters)
On‐chip	Cheng et al. Nat. Commun., 2024 [29]	On‐chip diffractive optics with heaters	5	80^b^	16 × 16	200 × 360 μm^2^	∼2400 mW (thermo‐optic phase shifters)
On‐chip	Xu et al. Science, 2024 [39]	Taichi photonic chiplet (hybrid)	4	4096	64 × 8	100 × 128 μm^2^	Passive for the diffractive module

^a^ The footprint of the free‐space configuration is defined as the area of a single diffractive layer, whereas for the on‐chip configuration, it is defined as the area of a single diffractive structure and its diffraction region.

^b^ The paper does not explicitly mention the number of neurons, so the data here are calculated based on the input/output channels and the number of layers.

## 3 Computational Capabilities: Reconfigurability and Multidimensional Modulation

While the structural evolution from free‐space to on‐chip and hybrid platforms establishes the physical foundation of DONNs, the architecture alone does not fully dictate the system's performance. To transition from static diffractive elements to intelligent computing engines capable of handling complex tasks, it is essential to equip these architectures with dynamic adjustability and high‐density information processing capabilities. Consequently, enhancing computational capabilities has become a central focus in the continued evolution of DONNs.

As illustrated in Figure 5, these capabilities are largely determined by two key factors: reconfigurability and multidimensional modulation, which play decisive roles in determining how flexibly and efficiently a DONN can perform optical computing tasks. In free‐space implementations, reconfigurability is mainly achieved through LC, SLM, and DMD devices [19, 21, 22, 27, 33, 34, 35, 36, 37, 38, 39, 40, 41, 42, 43, 44, 45, 46, 47, 48, 49], as well as electrically controlled meta‐atoms that enable programmable manipulation [28]. On‐chip platforms, by contrast, rely on thermo‐optic and electro‐optic phase shifters or all optical pumping schemes to achieve dynamic control [20, 26, 29, 32, 44]. Each approach offers unique advantages in terms of speed, scalability, and integration level. In parallel, recent advances in multidimensional modulation have extended the accessible optical degrees of freedom, allowing DONNs to exploit parameters such as space [60], time [27, 34, 48], twisting [36], direction [55, 64], frequency [26, 36], OAM [45, 50, 51], polarization [45, 47], and amplitude and phase [33, 38] encoding, either individually or in combination.

**FIGURE 5:**
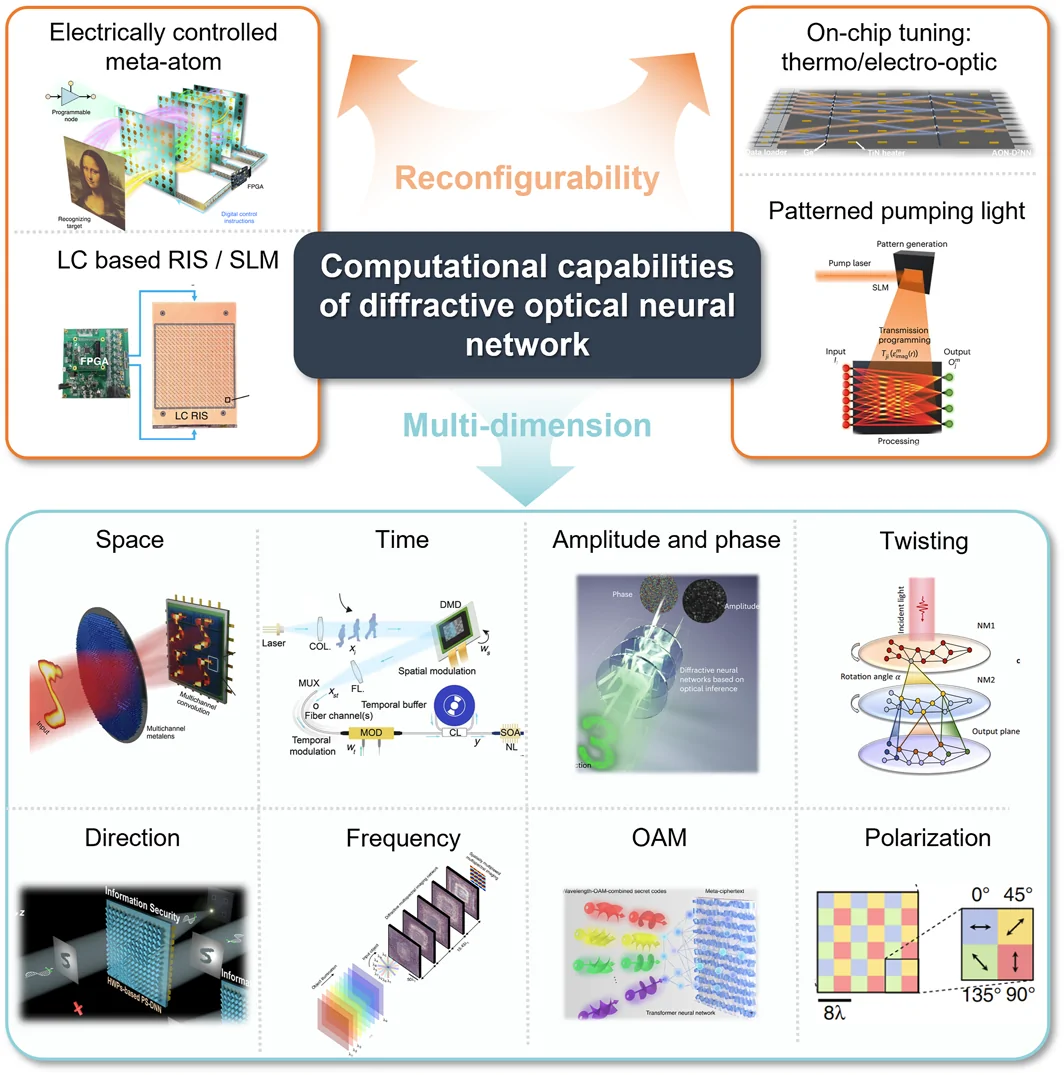
Overview of reconfigurable mechanisms and multidimensional modulation strategies that enhance the computational capabilities of DONNs. Reconfigurable approaches are categorized into free‐space and on‐chip implementations, whereas multidimensional computing is illustrated through eight distinct degrees of freedom. Electrically controlled meta‐atom. Reprinted with permission from Ref. [28]. Copyright 2022, Springer Nature. LC based RIS/SLM. Reprinted with permission from Ref. [27]. Copyright 2024, Springer Nature. On‐chip tuning: thermo/electro‐optic. Reprinted with permission from Ref. [44]. Under License CC BY. Patterned pumping light. Reprinted with permission from Ref. [26]. Copyright 2023, Springer Nature. Space. Reprinted with permission from Ref. [60]. Copyright 2022, The American Association for the Advancement of Science. Time. Reprinted with permission from Ref. [48]. Copyright 2022, The American Association for the Advancement of Science. Amplitude and phase. Reprinted with permission from Ref. [65]. Copyright 2025, Springer Nature. Twisting. Reprinted with permission from Ref. [36]. Copyright 2024, Springer Nature. Direction. Reprinted with permission from Ref. [55]. Copyright 2025, Springer Nature. Frequency. Reprinted with permission from Ref. [58]. Copyright 2023, Springer Nature. OAM. Reprinted with permission from Ref. [50]. Copyright 2025, The American Association for the Advancement of Science. Polarization. Reprinted with permission from Ref. [57]. Copyright 2022, Springer Nature.

These rich reconfigurability and modulation schemes significantly expand the functional capacity of DONNs, enabling them to tackle increasingly diverse and complex computational tasks. The following sections review in detail the representative strategies and implementations for reconfigurability and multidimensional optical modulation.

### 3.1 Reconfigurable Architectures: Optical and Electrical Implementations

As shown in Figure 6a, Wu et al. demonstrated a lithography‐free reconfigurable integrated photonic processor that achieves dynamic on‐chip programmability through optically induced gain–loss modulation [26]. Instead of using lithographically defined patterns, the device employs an unpatterned InGaAsP layer whose imaginary refractive index is locally tuned by a spatially structured pump beam. By adjusting the pump distribution in real time, the optical transmission matrix can be continuously reconfigured, enabling fully optical and reversible weight updates without additional fabrication. This work establishes a novel approach to all‐optical reconfigurable diffractive computing on an integrated photonic platform. In contrast to the optically driven approach of Wu et al., Cheng et al. realized an on‐chip reconfigurable diffractive optical neural network based on thermo‐optic phase tuning [29], as illustrated in Figure 6b. In this device, TiN microheaters integrated on a silicon‐on‐insulator platform locally modulate the refractive index through voltage‐controlled heating, enabling continuous adjustment of diffractive weights. This architecture allows direct in situ reconfiguration and training within the optical domain, demonstrating a representative electro‐thermal control mechanism for integrated DONNs.

**FIGURE 6:**
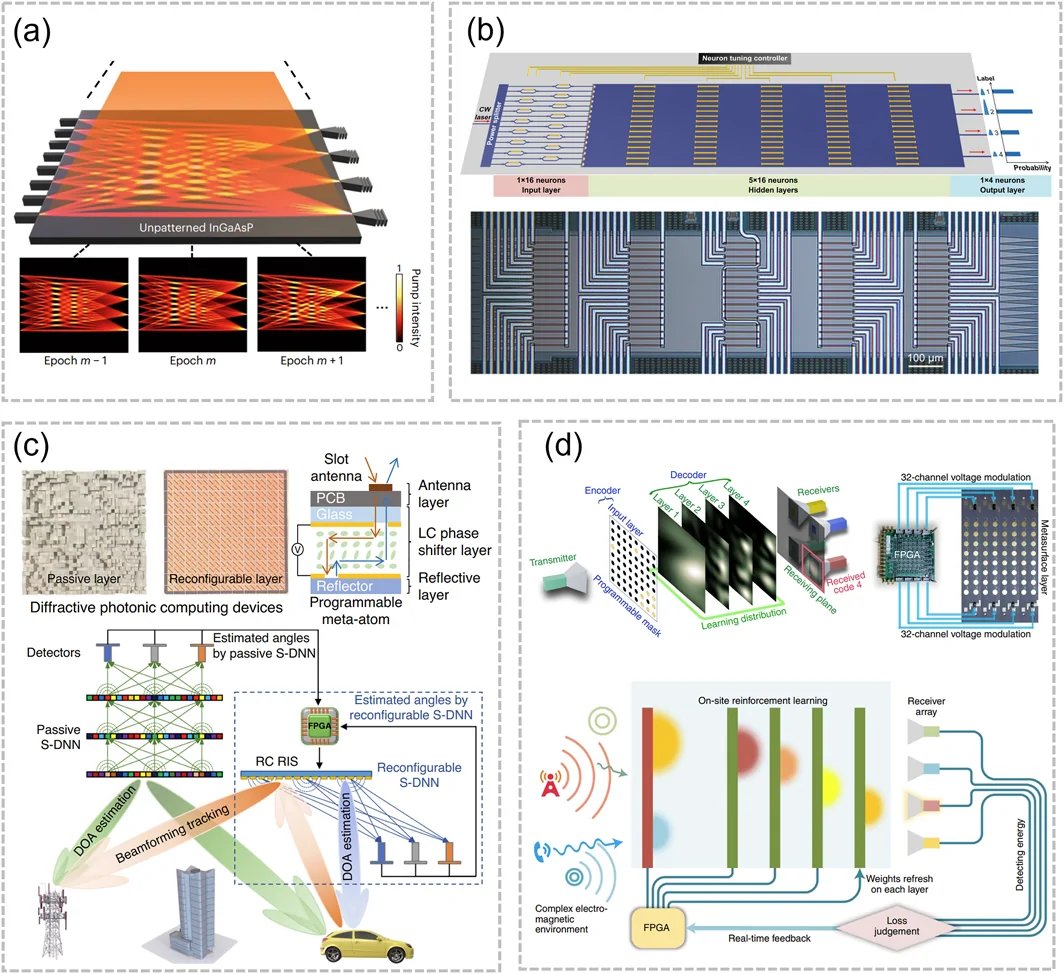
Representative reconfigurable DONN implementations across different platforms. (a) Lithography‐free reconfigurable on‐chip DONN, in which spatially programmable gain–loss profiles enable dynamic reconfiguration of diffractive neural operations. Reprinted with permission from Ref. [26]. Copyright 2023, Springer Nature. (b) Thermo‐optic modulation–based reconfigurable phase control on integrated chips, where the effective phase modulation of diffractive units is dynamically tuned via integrated microheaters to enable in situ reconfiguration of the network. Reprinted with permission from Ref. [29]. Copyright 2024, Springer Nature. (c) The upper left panel shows the structure of the passive diffractive layers, whereas the upper right panel presents the LC‐based reconfigurable diffractive neural network architecture. The lower panel displays the corresponding experimental results, demonstrating dynamic wave front manipulation enabled by the combination of passive diffractive layers and electrically tunable LC modulation. Reprinted with permission from Ref. [27]. Copyright 2024, Springer Nature. (d) Digital coding metasurface with voltage‐controlled meta‐atoms enabling dynamic phase modulation, where the upper panels show the multilayer digital coding metasurface architecture and programmable encoding/decoding operations, and the lower panel illustrates on‐site reinforcement learning for dynamic wave manipulation through real‐time feedback control. Reprinted with permission from Ref. [28]. Copyright 2022, Springer Nature.

For the free‐space implementations, Gao et al. realized a reconfigurable free‐space DONN by cascading a passive diffractive layer with an LC‐based RIS [27], as shown in Figure 6c. The LC RIS is an electronically addressable array of meta‐atoms driven by FPGA voltages that discretely tune the LC phase in reflection, enabling real‐time weight updates and temporal multiplexing between models. Working jointly with the passive layer, the system performs all‐optical direction‐of‐arrival estimation with angular super‐resolution and then feeds the estimates to the RIS for beamforming, establishing low‐latency communication links. Distinct from the LC RIS–based spatial reconfiguration, Liu et al. proposed a fully programmable diffractive deep neural network based on a multilayer digital coding metasurface array [28], as illustrated in Figure 6d. Each meta‐atom in the metasurface was integrated with two amplifier chips and controlled by an FPGA, enabling a tunable complex‐valued transmission coefficient over a 35‐dB dynamic range. Through this mechanism, both the amplitude and the phase of transmitted electromagnetic waves could be digitally reconfigured in real time, allowing on‐site training, weight updating, and task switching across diverse functions such as image recognition, multiuser communication decoding, and dynamic beam focusing.

Table 2 provides a quantitative comparison of the reconfigurable mechanisms discussed above, summarizing their reconfiguration speed, power consumption, modulation depth, modulation type, and platform compatibility. The choice of reconfiguration mechanism involves trade‐offs among speed, power efficiency, and system complexity, depending on the specific application requirements.

**TABLE 2:** Comparison of reconfigurable DONN approaches.

Reconfiguration mechanism	Ref.	Platform	Modulation parameter	Reconfiguration speed	Power consumption	Modulation depth/precision	In situ training
SLM	Chen et al. Nature, 2025 [19]	Free‐space	Phase	∼60 Hz (SLM)	∼1.9–3.5 W (SLM)	8‐bit SLM	/
Digital coding metasurface	Liu et al. Nat. Electron., 2022 [28]	Free‐space	Amplitude + phase	∼kHz (FPGA)	∼100 W (FPGA + amplifier node)	35 dB (from −22 to 13 dB)	Yes
DMD	Zhou et al. Sci. Adv., 2023 [48]	Free‐space	Amplitude	8024 Hz (SMUX‐STPC^a^)	/	1‐bit DMD (on/off)	No
LC‐based RIS	Gao et al. Light: Sci. Appl., 2024 [27]	Free‐space	Phase	> 2 Hz (LC response)	∼0.5 W (each LC RIS panel)	5‐bit FPGA	No
Thermo‐optic	Cheng et al. Nat. Commun., 2024 [29]	On‐chip	Phase	∼kHz (thermal response)	∼2400 mW (80 heaters)	16‐bit for programmable power (0–2π phase shift)	Yes
All‐optical pump	Wu et al. Nat. Photonics, 2023 [26]	On‐chip	Amplitude	∼10 Hz (carrier lifetime) ∼5 GHz (camera frame rate)	1.5 mW (optical pump)	−0.1 to 0.03 (imaginary part of refractive index)	Yes
All‐optical pump	Wu et al. Nat. Photonics, 2025 [32]	On‐chip	Amplitude	∼50 Hz (camera frame rate) ∼300 Hz (SLM)	0.32–1.53 mW (optical pump)	/	Yes
Electro‐optic	Wang et al. Nat. Photonics, 2025 [20]	On‐chip	Phase	∼5 MHz (peripheral equipment bandwidth)	/	8‐bit for programmable power	No

^a^ SMUX‐STPC represents the spatial multiplexing spatiotemporal photonic computing system.

### 3.2 Training Strategies: From Offline Optimization to In Situ Learning

The reconfigurable mechanisms discussed in Section 3.1 not only enable multitask operation and adaptive inference, but also open the door to the in situ learning. By allowing real‐time adjustment of diffractive weights, reconfigurable DONNs can be optimized directly on the physical hardware, thereby overcoming the limitations inherent to purely simulation‐based training. This section reviews the training strategies for DONNs, starting with conventional offline approaches and then focusing on emerging in situ training methods that leverage the reconfigurability of modern DONN implementations.

#### 3.2.1 Offline Training and Simulation‐to‐Reality Gap

Offline training follows the conventional deep learning paradigm, where the DONN is modeled as a differentiable computational graph and optimized using gradient‐based methods. The forward propagation through each diffractive layer is simulated using the Rayleigh–Sommerfeld diffraction integral or its efficient approximation via the angular spectrum method [88]. Because all operations are differentiable, the gradients of the loss function with respect to the learnable parameters can be computed analytically using the chain rule and back‐propagated through the network [35].

However, a fundamental challenge associated with offline training is the simulation‐to‐reality gap, which arises from multiple sources, including fabrication imperfections, optical misalignment between layers, material dispersion and absorption, environmental perturbations, and detector noise. Consequently, the experimental accuracy of offline‐trained DONNs often falls significantly below the simulated performance. Several strategies have been developed to mitigate the simulation‐to‐reality gap within the offline training framework. Physics‐aware training incorporates known physical imperfections into the forward simulation model by adding random phase noise, Gaussian blur, or other perturbation models during training, thereby improving robustness to real‐world imperfections [89]. Data augmentation techniques, including geometric transformations and noise injection on the input images, enhance the generalization capability of the trained network [90].

#### 3.2.2 In Situ Training: Principles and Implementations

In situ training fundamentally addresses the simulation‐to‐reality gap by performing the optimization process directly on the physical hardware, thereby automatically accounting for all system imperfections without requiring explicit modeling. The core principle is to measure the actual optical output of the physical DONN and use this information to update the tunable parameters, ensuring that the optimization converges to a configuration that is optimal for the real system rather than an idealized simulation. The implementation of in situ training requires reconfigurable DONNs whose parameters can be dynamically adjusted, as discussed in Section 3.1. Several representative approaches have been demonstrated in recent years.

Zhou et al. demonstrated an adaptive training approach for a reconfigurable diffractive processing unit based on DMD and SLM [22]. The training process begins with conventional in silico pre‐training, after which the model is transferred to the physical optoelectronic system. To address the accumulated system errors that cause performance degradation, the physical outputs of each layer are experimentally recorded using samples from the training set, and these measured outputs are then used as inputs for in silico retraining of the subsequent layers. Although the above approach performs fine‐tuning in simulation using physically measured intermediate outputs, more advanced methods have been developed that employ learnable digital models to continuously track and adapt to the physical system's behavior throughout the training process.

A more sophisticated class of in situ training methods employs learnable digital models that continuously adapt to represent the physical system's behavior. Zheng et al. proposed a dual adaptive training framework [21], where a digital proxy model is jointly optimized alongside the physical DONN. The digital model is continuously updated to minimize the discrepancy between its predictions and the measured physical outputs, and the gradients for updating the physical parameters are computed through this adaptive model. Similarly, Yuan et al. introduced a dual‐neuron optical artificial learning framework [47], where each physical optical neuron is paired with a digital artificial neuron that learns to model its input–output behavior. This hybrid strategy effectively combines the fidelity of physical measurements with the computational efficiency of digital backpropagation.

The aforementioned in situ training approaches have been primarily demonstrated in free‐space implementations using SLMs or DMDs as reconfigurable elements. For on‐chip DONNs, in situ training has also been explored using integrated tunable components. Cheng et al. demonstrated an on‐chip diffractive optical neural network with in situ training capability on a silicon‐on‐insulator platform [29]. The system employs microheaters to provide thermo‐optic phase tuning for each unit. During in situ training, the network parameters are updated based on measured optical outputs, allowing the system to learn directly from physical measurements and inherently compensate for fabrication variations and environmental perturbations.

### 3.3 Multidimensional Optical Multiplexing

In addition to reconfigurability, enhancing the computational capacity of DONNs also relies heavily on exploiting the intrinsic multidimensional degrees of freedom of light. Recent studies have demonstrated diverse forms of multiplexed diffractive computing with enriched functionality and scalability. Representative works across different optical degrees of freedom are summarized in the following examples.

Among the various optical degrees of freedom, amplitude and phase modulation represent the most fundamental and widely adopted strategies in DONNs. These two parameters directly determine the complex‐valued transmission function of each diffractive unit and thus form the physical basis for feature transformations. As illustrated in Figure 7a, Yu et al. demonstrated a representative example utilizing both amplitude and phase multiplexing for enhanced optical information encoding [65]. In their design, a miniaturized diffractive neural network was integrated on the end facet of a multimode fiber, where the diffractive layers were engineered to jointly manipulate the amplitude and the phase of the transmitted field. By coordinating these two modulation dimensions, the network effectively compensated for modal dispersion and cross talk during multimode propagation, achieving high‐fidelity image transmission and reconstruction. As shown in Figure 7b, Guo et al. integrated polarization conversion and propagation direction selectivity within a cascaded metasurface framework [55]. By combining quarter‐wave or half‐wave plate arrays with metallic gratings, the system achieves polarization‐controlled switching between unidirectional and bidirectional operation modes, thereby realizing both information security (one‐way propagation) and information sharing (two‐way propagation). This design effectively exploits two independent degrees of freedom for multiplexed optical processing and data encoding.

**FIGURE 7:**
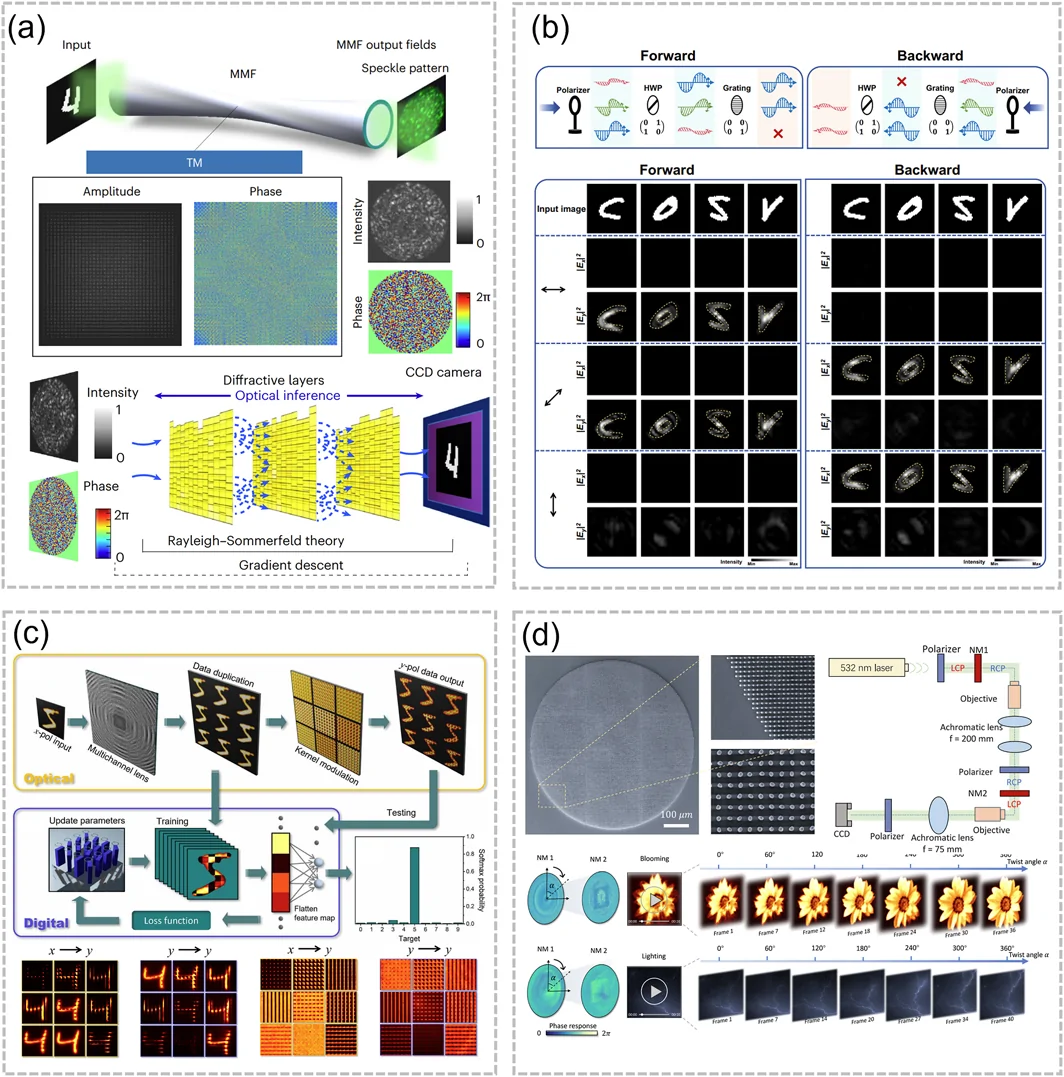
Representative multidimensional modulation strategies in DONNs. (a) Simultaneous amplitude and phase modulation for enhanced information encoding and efficient optical computation. The top panel shows the complex speckle patterns generated by a multimode fiber, whereas the bottom panel depicts the training of diffractive layers to jointly manipulate the amplitude and the phase for image reconstruction. Reprinted with permission from Ref. [65]. Copyright 2025, Springer Nature. (b) Polarization and direction‐dependent diffractive processing enabling multichannel optical inference. The top and bottom panels, respectively, show the schematic and the experimental demonstration of the working principle of the unidirectional–bidirectional–unidirectional DONN under *x*‐, 45°‐, and *y*‐polarized THz illumination from the forward and backward directions. Reprinted with permission from Ref. [55]. Copyright 2025, Springer Nature. (c) Combination of polarization and spatial degrees of freedom to improve data throughput. A multichannel metalens spatially duplicates the input, while a polarization‐sensitive kernel layer performs parallel convolutions for object classification. Reprinted with permission from Ref. [60]. Copyright 2022, The American Association for the Advancement of Science. (d) Twist‐controlled diffractive network leveraging polarization and twist coupling for multidimensional learning. The top panel displays image retrieval via mechanical rotation of a meta‐disk, whereas the bottom panel illustrates multichannel decoupling utilizing the twist degree of freedom. Reprinted with permission from Ref. [36]. Copyright 2024, Springer Nature.

Similarly leveraging the polarization degree of freedom, Zheng et al. further combined it with spatial multiplexing to enhance parallel processing in DONNs [60], as illustrated in Figure 7c. They proposed a meta‐optic accelerator composed of two cascaded metasurfaces: a multichannel metalens that spatially divides the incident light into nine sub‐images and a polarization‐sensitive kernel layer that performs vector matrix multiplication. Each meta‐atom's rotation angle defines polarization conversion and amplitude weighting, enabling simultaneous processing of multiple spatial channels and polarization states. This hybrid use of polarization and spatial degrees of freedom allowed concurrent classification of image content and polarization information with high accuracy. Introducing the twist degree of freedom as a new dimension for optical multiplexing, Fan et al. developed a twisted diffractive neural network that encodes and processes information through controlled interlayer rotation of metasurfaces [36], as shown in Figure 7d. Each layer of the stacked Pancharatnam–Berry metasurface is rotated by a defined angle, where the twist angle modulates the coupling between diffraction channels, giving rise to distinct optical transfer paths and interference patterns. This rotational configuration naturally couples with the polarization‐dependent geometric phase, enabling multiplexed control of polarization and twisting within a single device.

Expanding beyond previously explored dimensions, Meng et al. introduced wavelength and OAM into DONN design [50]. As shown in Figure 8a, they realized an ultranarrow‐linewidth wavelength‐vortex multiplexing metasurface hologram by engineering the dispersion of k‐vector components and combining wavelength‐dependent grating functions with OAM phase encoding. A sparse k‐vector filtering aperture array was designed in momentum space to ensure sharp wavelength selectivity while maintaining distinct OAM channels. Leveraging transformer neural networks to optimize phase‐only holograms, the metasurface achieved simultaneous reconstruction of over one hundred independent images with a linewidth as narrow as 2nm. This hybrid control of wavelength and OAM greatly expands the information capacity and functional diversity of diffractive optical systems.

**FIGURE 8:**
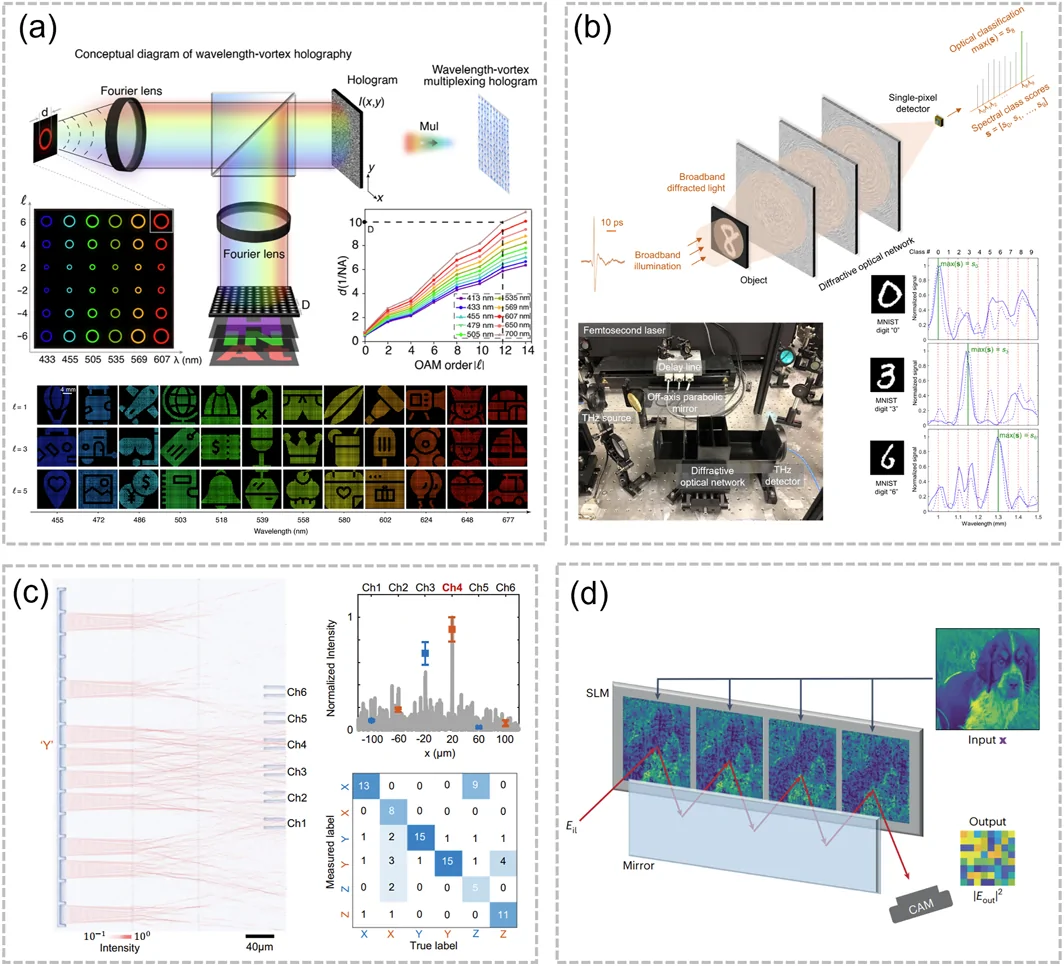
Representative multidimensional modulation strategies in DONNs. (a) Joint utilization of OAM and wavelength for multichannel optical inference. A sparse k‐vector filtering aperture array in momentum space is employed to simultaneously achieve the wavelength and OAM selectivity for ultranarrow‐linewidth holographic multiplexing. Reprinted with permission from Ref. [50]. Copyright 2025, The American Association for the Advancement of Science. (b) Spectrally encoded single‐pixel machine vision. The system utilizes wavelengths to encode input spatial information, which is detected by a single‐pixel detector. The frequency corresponding to the highest spectral component identifies the final classification result. Reprinted with permission from Ref. [62]. Copyright 2021, The American Association for the Advancement of Science. (c) On‐chip DONN integrating wavelength multiplexing for compact and parallel optical processing. The integrated metasystem employs dispersion‐engineered metasurfaces to spatially map different input wavelengths into distinct parallel output waveguides. Reprinted with permission from Ref. [37]. Copyright 2022, Springer Nature. (d) Temporal domain DONN utilizing pulsed light modulation for high‐speed information encoding and processing. The physical implementation of nonlinear processing with only linear optics, featuring a single‐pass multi‐bounce cavity configuration. Reprinted with permission from Ref. [34]. Copyright 2024, Springer Nature.

Although the previous work exploited wavelength multiplexing, Fang et al. demonstrated a distinct approach that directly encodes information in the wavelength domain for single‐pixel machine vision. As shown in Figure 8b, they designed a diffractive optical processor that converts spatial information into unique spectral responses through wavelength‐selective diffraction [62]. A single‐pixel detector then captures these spectral signatures, which are decoded by a neural network to perform object recognition without spatial imaging. This method uses pure spectral multiplexing, but unlike previous multichannel schemes, it achieves dimensional reduction by converting spatial features into wavelength‐encoded spectra, representing a compact and energy‐efficient route for DONNs. In addition to the free‐space implementations of wavelength multiplexing discussed above, on‐chip implementations have also been developed to integrate spectral control within compact photonic platforms. As shown in Figure 8c, Wang et al. demonstrated an integrated photonic metasystem that performs wavelength‐dependent diffractive computing directly on a silicon‐on‐insulator chip [37]. The device integrates multiple metasurface layers to achieve dispersion‐engineered diffraction, enabling distinct optical mappings at different wavelengths. Parallel output waveguides provide on‐chip spectral demultiplexing, realizing all‐optical wavelength‐dependent computation. This work combines the compactness of on‐chip photonics with the spectral selectivity of DONNs.

Temporal domain modulation provides another effective dimension for expanding the computational space. As shown in Figure 8d, Yildirim et al. proposed a nonlinear processing with only linear optics framework [34], which realizes dynamic optical computation through time‐sequenced data repetition within a multi‐bounce optical cavity. Using an LC spatial light modulator and a mirror, multiple modulation planes are formed within a single round trip, allowing the same optical field to interact with successively encoded temporal layers. This design enables nonlinear transformations and learnable polynomial mappings using low power continuous wave light, effectively translating temporal repetition into enhanced optical nonlinearity.

While the multidimensional modulation strategies discussed above significantly expand the functional capacity of DONNs, it is important to note the inherent trade‐offs among different optical degrees of freedom. In terms of information capacity, wavelength multiplexing can readily scale to tens or even hundreds of channels leveraging mature WDM technologies, whereas OAM‐based approaches offer theoretically unlimited orthogonal modes but face practical limitations in mode purity and cross talk. Polarization provides only a limited number of orthogonal states but offers robust and straightforward implementation. Regarding scalability, spatial and wavelength multiplexing are generally more amenable to large‐scale parallelization, whereas OAM and twist‐based approaches require increasingly precise alignment and fabrication as the mode order or the twist angle increases. From the perspective of fabrication complexity, amplitude and phase modulation rely on well‐established lithographic or 3D‐printing techniques, whereas polarization and OAM‐sensitive designs demand sub‐wavelength geometric control of meta‐atoms. The selection of appropriate modulation dimensions should therefore be guided by specific application requirements, balancing information throughput, system scalability, and practical fabrication constraints.

In summary, the computational capabilities of DONNs have been advanced mainly through reconfigurability and multidimensional modulation. Reconfigurable mechanisms enable dynamic control of optical weights for adaptive or multitask operation, while multidimensional modulation leverages various optical degrees of freedom to expand information channels and functional diversity. Together, these strategies transform DONNs from static diffractive systems into adaptive, high‐dimensional photonic processors capable of more versatile and scalable optical computing.

## 4 Functional Applications of DONNs

DONN's multidimensional modulation ability and flexible reconfigurability enable it to have functional applications in many aspects. Spatially configured DONNs enable direct wave front manipulation of input light, allowing them to natively process high‐dimensional (2D/3D) data. This capability makes them particularly suitable for image processing applications [18] and beam shaping tasks [91]. Meanwhile, on‐chip architectures offer scalable solutions for large‐scale computational problems [19]. DONNs can also facilitate logical operations [69, 92] and communication tasks [33], serving as a cornerstone for developing all‐optical computing. Furthermore, the recent advancement of quantum information technologies has opened new avenues for deploying DONNs in quantum computing systems [93, 94].

### 4.1 Image Processing

DONNs have demonstrated considerable potential in image processing, with applications spanning imaging [83, 95], image classification [93, 94, 96, 97, 98, 99, 100, 101, 102, 103, 104, 105, 106, 107, 108, 109] [110, 111, 112], specialized image transformation [113, 114, 115, 116, 117, 118, 119, 120, 121, 122], and generative tasks [19, 20, 39, 46]. Several representative works in this domain are illustrated in Figure 9.

**FIGURE 9:**
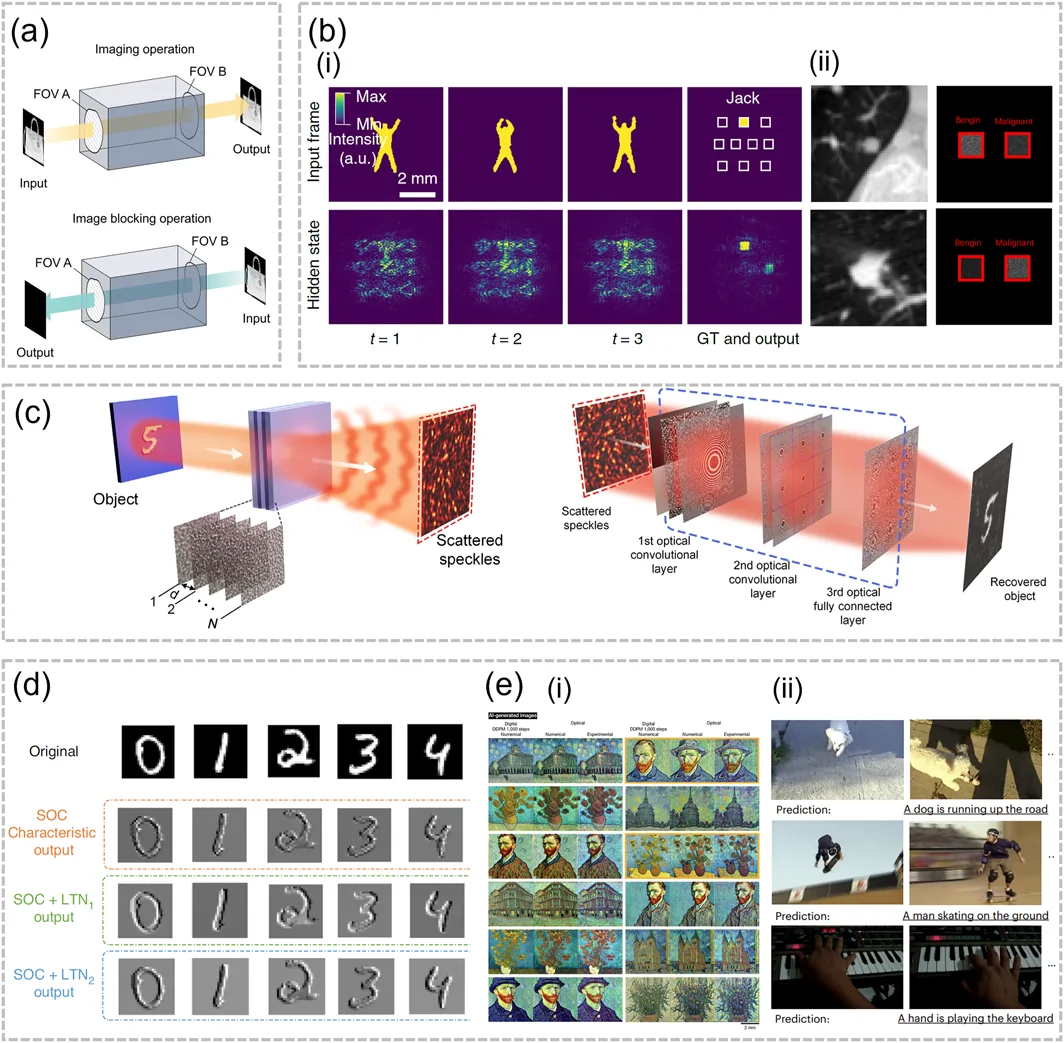
Applications of DONN related to image processing. (a) One‐way imaging. This system permits high‐fidelity image formation in the forward direction contrarily blocking information retrieval in the reverse path through scattering. Reprinted with permission from Ref. [64]. Copyright 2023, The American Association for the Advancement of Science. (b) Input images and classification results done by DONNs. (i) Human action recognition. Reprinted with permission from Ref. [22]. Copyright 2021, Springer Nature. (ii) Pulmonary nodule classification. Reprinted with permission from Ref. [113]. Under License CC BY 4.0. (c) Schematics of memory‐less scattering imaging. The input digit generates a scattered speckle pattern through a scattering medium, which is subsequently processed by the diffractive network to reconstruct the original image [68]. Reprinted with permission from Ref. [68]. Copyright 2024, The American Association for the Advancement of Science. (d) Realization of image transformation tasks. The panel displays a subset of the experimental results, showing feature maps of MNIST handwritten digits produced by the shared optical core (SOC), SOC + lightweight transfer network (LTN) 1, and SOC + LTN2, respectively. Reprinted with permission from Ref. [71]. Copyright 2025, Springer Nature. (e) Results of generating models driven by diffractive units. (i) Results of a multicolor optical generative model for colorful Van Gogh–style artwork generation. Reprinted with permission from Ref. [19]. Copyright 2025, Springer Nature. (ii) A visualization of the sequence of optical inputs sampled from original videos and their corresponding captions recognized by a well‐trained diffractive model. Reprinted with permission from Ref. [20]. Copyright 2025, Springer Nature.

As shown in Figure 9a, a DONN was designed in Reference [64] to perform unidirectional imaging, allowing light transmission and image formation from one direction and completely blocking the reverse path on the other hand. Similarly, a pyramid DONN was demonstrated in Reference [101] for asymmetric image magnification and demagnification. These works exemplify the unique capability of DONNs in achieving optical functions that are difficult to realize with conventional systems.

As a neural network–based architecture, DONN finds a major application in image classification. Its inherent structure enables efficient classification tasks with minimal preprocessing of input images. Numerous studies have validated their DONN architectures using standard image recognition datasets such as MNIST [123]. Zhou et al. demonstrated the capability to classify dynamic objects by processing multiple video frames, as illustrated in Figure 9bi [22]. The versatility of DONNs in handling diverse image classification tasks is further evidenced by their application in pulmonary nodule detection and classification, presented in Figure 9bii [113].

DONNs are also well suited for image transformation tasks, including encoding/decoding and concealment/reconstruction, owing to their inherent compatibility with 2D image data and high‐speed processing capabilities. As shown in Figure 9c, Zhang et al. realized memory‐less scattering imaging and reconstruction using a convolutional DONN that operates beyond the conventional optical memory effect [68]. In another example, Bai et al. demonstrated information concealment by encoding object data into the pattern of a digit ‘8' via a DONN, with successful subsequent reconstruction. With appropriate design, on‐chip DONN can accomplish diverse image processing tasks and even support novel functionalities. Although on‐chip configurations cannot directly process planar information as free‐space architectures do, they are exceptionally well suited for handling large‐volume input in batch‐processing mode. As illustrated in Figure 9d, an integrated multitask on‐chip design simultaneously performs image edge detection and classification [71].

Furthermore, the superior efficiency and reconfigurability of DONNs enable their application to large‐scale generative modeling tasks. As illustrated in Figure 9ei, vibrant Van Gogh‐style artworks have been generated using SLMs coupled with diffractive decoders [19]. In a more advanced implementation, Reference [20] demonstrated a diffractive tensorized optical network capable of executing complex generative tasks, including video caption prediction as shown in Figure 9eii. In summary, the application of DONNs in image processing presents a highly promising and exciting research direction.

### 4.2 Beam Shaping and Multiplexing

Beyond image processing, DONNs offer versatile wave front manipulation capabilities that extend to the direct control of light beams themselves. These include spatially shaping optical fields [69, 91, 124, 125], temporally tailoring pulse profiles [126], managing OAM states [51, 80, 127], and controlling guided modes in integrated photonic structures [124, 128]. Representative implementations of these functionalities are respectively illustrated in Figure 10.

**FIGURE 10:**
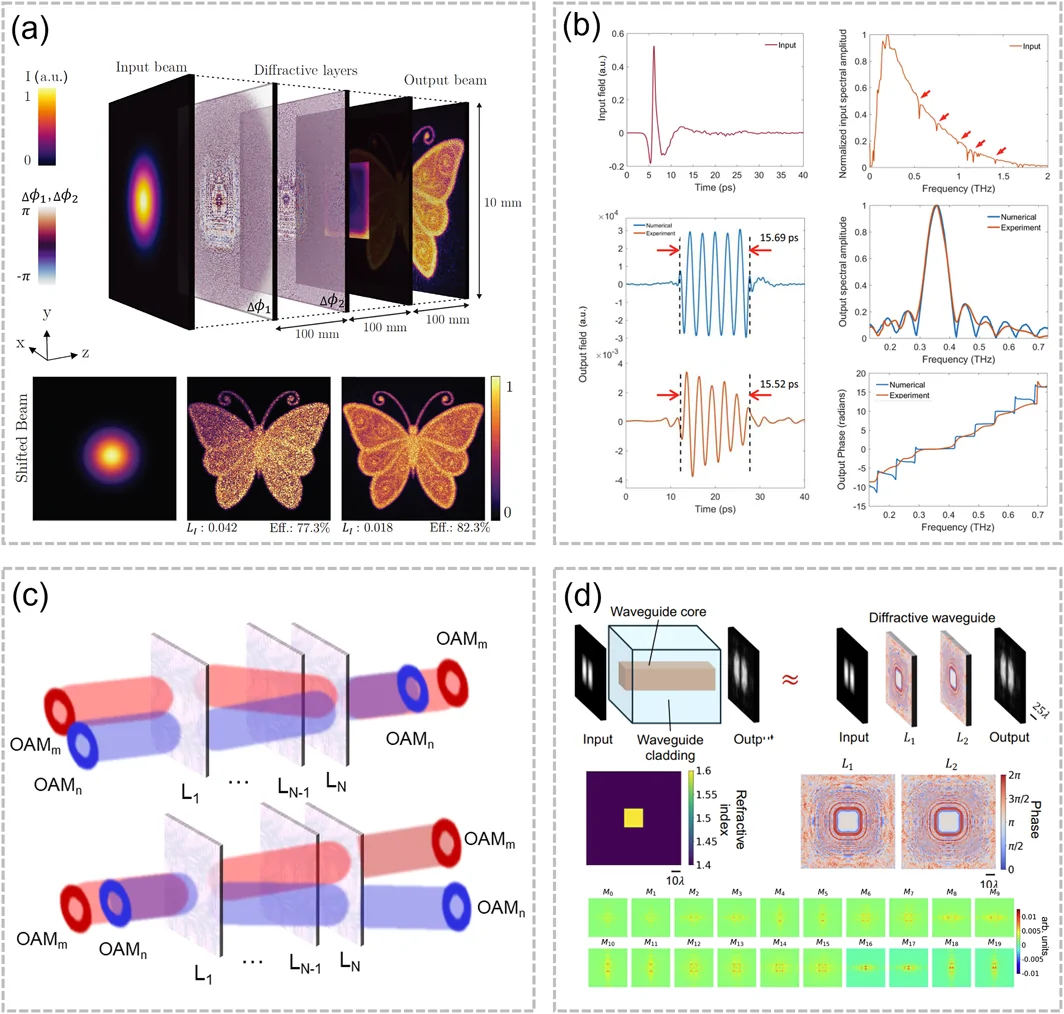
Schematics of beam shaping and multiplexing tasks accomplished by DONNs. (a) Laser beam shaping. The multilayer diffractive system modulates the input beam phase through cascaded elements to generate a designated intensity profile at the target plane. Reprinted with permission from Ref. [125]. Under License CC BY 4.0. (b) Terahertz pulse shaping. The diffractive network synthesizes a targeted temporal waveform by independently modulating the amplitude and the phase of the broadband input spectrum. The panels display the time‐domain pulse envelope and the corresponding spectral amplitude and phase distributions. Reprinted with permission from Ref. [126]. Copyright 2021, Springer Nature. (c) DONN for OAM multiplexing and demultiplexing. An N‐layer network is used to couple (top panel) and separate (bottom panel) the VBs with different OAM modes. (d) Optimized diffractive structure performing as dielectric waveguide. The left panel shows the cross section of a traditional square dielectric waveguide consisting of core and cladding dielectric regions, whereas the right panel shows the schematic of a cascadable diffractive waveguide unit designed to match the performance of the square dielectric waveguide. The bottom panel shows the error of the output fields from the two waveguides. Reprinted with permission from Ref. [128]. Copyright 2025, Springer Nature.

As demonstrated in Figure 10a, Reference [125] utilized a DONN to transform a laser beam into a butterfly‐shaped intensity profile, with the output pattern remaining clearly defined even with the bottom beam shifted. As another essential beam shaping task, focusing has also been advanced using DONNs, enabling effective focusing of beams at large incident angles [86]. Beyond spatial wave front control, DONNs can also manipulate light in the temporal domain. Figure 10b presents results of terahertz pulse shaping achieved via diffractive surfaces [126].

The wave front–shaping capability of DONNs enables effective manipulation of OAM states, facilitating diverse applications in structured light processing. As schematically illustrated in Figure 10c, a DONN‐based system performs optical OAM multiplexing and demultiplexing, enabling mode division multiplexing with high mode purity, low error rate in communication, all‐optical information processing, etc. [127]. Beyond multiplexing, Reference [51] demonstrated image encoding and decoding through OAM mode projection using a DONN architecture. The same framework has also been extended to OAM‐based classification tasks, where different data classes are mapped to distinct OAM modes for all‐optical inference [45].

DONNs further provide a versatile platform for controlling guided modes in waveguide‐based systems. As shown in Figure 10d, an optimized DONN can match the performance of conventional dielectric waveguides while enabling advanced functional operations [128]. In a related implementation, Reference [124] demonstrated high‐efficiency ring‐core fiber coupling through precise tailoring of an optical vortex beam using a DONN. These examples underscore the potential of DONNs as powerful and adaptive tools for various tasks corresponding to light beam optimization.

### 4.3 Logic Operation, Communication, and Quantum Computing

DONNs have expanded their applicability to broader computational domains, including logic operations and optical communication. Furthermore, preliminary research into their potential compatibility with quantum optical systems suggests potential for future deployment in quantum computing architectures.

Optical logic gates implemented in free‐space DONNs operate on a principle analogous to image classification: Input signals are spatially encoded, and subsequent diffractive layers scatter the light toward predefined regions at the output plane to represent logical outcomes [76]. When properly designed, such gates like optical NAND units can be cascaded by optically connecting the output of one stage to the input of the next, enabling the execution of complex logic functions [96]. Beyond free‐space implementations, on‐chip DONN have also been successfully employed for logic gate realization, offering a route toward practical integration [85]. Moreover, DONN‐based logic can be extended to polarization‐domain processing, as demonstrated in Reference [95] using linearly polarized optical modes. Given the foundational importance of logic gates in general purpose computing, these developments highlight the significant potential of DONNs in building all‐optical logic circuits and advancing novel forms of optical computing.

DONNs also offer a versatile platform for advancing optical communication systems across multiple domains. Key communication–oriented functionalities such as information compression [129], data concealment [52], and optical encoding/decoding [99] have been successfully demonstrated using these architectures. In fiber‐based systems, DONNs enable efficient mode generation [124] and mode division de/multiplexing [127], enhancing data transmission capacity. For wireless applications, real‐time programmable DONNs constructed with RIS can directly modulate information onto electromagnetic carriers or demodulate incoming signals, providing a reconfigurable physical‐layer solution for adaptive wireless links [97]. These capabilities position DONNs as an enabling technology for next‐generation optical and wireless communication architectures.

Quantum computing has garnered significant research attention in recent years, and DONNs have demonstrated notable potential for implementing specific quantum computational operations. Both the polarization states and spatial modes of light can serve as carriers for quantum states, and DONNs have proven capable of effectively manipulating these optical degrees of freedom, making them suitable for quantum computing applications. In the work by Ding et al., linear polarization states were used to represent the |0〉 and |1〉 basis states, with a DONN successfully implementing four fundamental quantum gates: the Pauli‐X, Pauli‐Y, Pauli‐Z, and Hadamard gates. By illuminating different regions of the metasurface, all these operations were reliably achieved [94]. In Reference [93], three Laguerre–Gaussian modes served as the input and output states, enabling the realization of a high‐dimensional DONN‐based quantum gate. As can be seen, the application of DONNs continues to be actively explored in these emerging domains.

## 5 Discussion and Outlook

DONNs have emerged as a promising paradigm in optical computing, bridging optical physics and machine learning to realize large‐scale, high‐throughput, and energy‐efficient information processing. From early free‐space demonstrations based on 3D‐printed diffractive layers to recent integrated and hybrid configurations, the field has progressed rapidly toward greater functionality and compactness. The fundamental principle states that using engineered diffractive structures to perform linear transformations of light fields has evolved from static inference systems to dynamically tunable and multidimensional photonic computing platforms.

This review has summarized the evolution of DONNs from structural, computational, and functional perspectives. Architecturally, two major implementation routes have formed: free‐space implementations that offer massive neuron scalability and on‐chip implementations that emphasize miniaturization and integration. Hybrid configurations that combine diffractive elements with interferometric or resonant photonic circuits further expand the design space, enabling high‐density and multifunctional computing. These implementations collectively establish a foundation for optically driven computation that can be tailored to diverse applications in imaging, sensing, and communications.

Enhancing the computational capability of DONNs has become a central focus in recent years. Reconfigurability and multidimensional modulation represent two key directions driving this progress. Reconfigurable mechanisms span all‐optical, thermal, and electrical mechanisms, allowing direct tuning of optical weights and in situ retraining, transforming static diffractive layers into adaptive networks. Meanwhile, multidimensional modulation strategies leverage the rich physical degrees of freedom of light, including amplitude, phase, polarization, space, wavelength, OAM, twist, direction, and time. The coordinated exploitation of these freedoms enables parallel information channels and richer feature encoding, extending DONNs toward high‐dimensional, adaptive photonic processors.

Despite remarkable progress, several challenges remain before DONNs can reach practical and scalable deployment. The first challenge is training and generalization. The current design pipeline still relies heavily on numerical training followed by physical fabrication, often with a mismatch between simulated and experimental performance. Developing hybrid electronic–optical training frameworks, physics‐aware optimization algorithms, and in situ learning schemes will be essential for robust adaptation under real‐world perturbations. The second challenge is reconfigurability and efficiency trade‐off. Actively tunable components often introduce power overhead, slower response time, or limited modulation depth. Future materials together with device‐level innovations are expected to balance reconfiguration speed, stability, and energy consumption. The third challenge is that although free‐space implementations have demonstrated rich combinations of optical degrees of freedom for parallel optical processing, on‐chip implementations remain largely limited to phase, amplitude, or wavelength modulation. The integration of multiple optical degrees of freedom within a compact photonic platform is still challenging. Developing multidimensional sensitive diffractive units, as well as hybrid coupling schemes between metasurfaces and integrated waveguides, will be essential to bring the functional richness of on‐chip systems to a level comparable with their free‐space counterparts. The fourth challenge is scalability boundaries. Although DONNs are frequently highlighted for their massive scalability, their practical implementations face quantifiable physical limits that warrant careful analysis. In free‐space systems, the pioneering demonstration by Lin et al. employed 5 diffractive layers with a total of 0.2 million neurons (approximately 200 × 200 = 40,000 per layer) and around 8 billion connections [18]. Subsequent simulations showed that increasing to 10 layers and 0.4 million neurons could improve classification accuracy [18], and recent visible wavelength implementations using LC retarders have reached 1 million neurons per layer (1000 × 1000 arrays) over 5 layers [105]. More recently, Chen et al. demonstrated LightGen [23], an all‐optical generative chip integrating 6 diffractive metasurface layers with up to 400 × 400 photonic neurons per layer, achieving approximately 2.16 million neurons within a compact footprint of 136.5 mm^2^. This represents the deepest experimentally demonstrated free‐space DONN architecture to date, and most experimental work stays at 3–5 layers. Although free‐space DONNs can in principle be scaled to tens or even hundreds of millions of neurons using large‐area fabrication methods [40], the number of layers remains practically constrained to approximately 6 in current experimental demonstrations, as accumulating diffraction loss, interlayer alignment errors, and optical aberrations progressively degrade signal fidelity with increasing depth. For on‐chip implementations, Fu et al. demonstrated a three‐hidden‐layer DONN with 186 neurons per layer within a footprint of only 0.3 mm^2^ [42]. The trainable DONN chip by Cheng et al. integrated 5 hidden layers with 80 thermo‐optic tunable neurons [29]. At a larger scale, the Taichi chiplet achieved 13.96 million neurons through a distributed diffractive‐interference hybrid architecture [39]. Despite these advances, on‐chip scalability still faces several fundamental constraints: The total neuron count is bounded by the chip area and lithographic resolution; an increase in the network width introduces fan out penalties as optical power is distributed among a growing number of output channels; and cross talk arising from evanescent coupling between closely spaced waveguides or thermal interference between adjacent tunable elements, as well as mode mixing in multimode slab regions, impose practical limits on integration density. These factors collectively define the current scalability envelope of DONNs and underscore the need for systematic co‐optimization of optical design, fabrication technology, and network architecture to push toward larger and deeper diffractive computing systems. The fifth challenge is nonlinearity and hybrid computing. Linear optical transformations limit computational expressiveness. Combining optical front ends with electronic or optoelectronic nonlinear units or realizing optical nonlinearities via material or cavity dynamics could bring DONNs closer to the functional richness of digital deep networks [130, 131].

In conclusion, DONNs have evolved from conceptual optical processors to a versatile class of photonic computing architectures capable of adaptive, multidimensional, and scalable operation. As materials, fabrication, and algorithmic techniques continue to mature, DONNs are poised to play an increasingly central role in the next generation of intelligent photonic computing systems, paving the way toward intelligent and scalable all‐optical computation.

## Author Contributions

All authors have accepted responsibility for the entire content of this manuscript and approved its submission.

## Funding

This work was supported by the National Key Research and Development Program of China (Grant 2024YFA1209204), the National Natural Science Foundation of China (Grants 12474322 and 12504428), the Quantum Science and Technology‐National Science and Technology Major Project (Grant 2021ZD0301500), and the Postdoctoral Fellowship Program and China Postdoctoral Science Foundation (Grant BX20250171).

## Conflicts of Interest

The authors declare no conflicts of interest.

## Data Availability

Data sharing is not applicable to this article as no datasets were generated or analyzed during the current study.
